# Acute poisoning death resulting from a veterinary employee’s isoflurane abuse

**DOI:** 10.1007/s12024-025-01121-5

**Published:** 2025-10-29

**Authors:** Ako Sasao, Kyoko Hirata, Kosei Yonemitsu, Yuki Ohtsu, Hiroshi Tsutsumi, Shota Furukawa, Rie Sano, Yoko Nishitani

**Affiliations:** 1 Department of Forensic Medicine, Faculty of Life Sciences, Kumamoto University, Kumamoto, Japan; 2 Department of Forensic Medicine, Kyoto University Graduate School of Medicine, Kyoto, Japan

**Keywords:** Isoflurane, Gas chromatography-mass spectrometry, Postmortem distribution, Veterinary clinics, Anesthetic abuse, Case report

## Abstract

Isoflurane is a volatile inhalation anesthetic used primarily in veterinary medicine and, recently, infrequently in human medical applications. However, it is sometimes abused for recreational or suicidal purposes by medical and research personnel. We present a forensic autopsy case of a man in his 40 s who was employed at a veterinary hospital and was suspected to have died from isoflurane poisoning. Autopsy findings showed no injuries or lesions, and the pathological findings of most organs suggested acute death. Routine gas chromatography–mass spectrometry analysis for blood alcohol showed no ethanol, but showed a peak that was identified as isoflurane. Quantitative analysis of isoflurane in blood and tissues was performed by gas chromatography-mass spectrometry. The isoflurane concentrations in the left and right heart blood and femoral blood were 49.8, 54.6, and 44.7 µg/mL, respectively. These concentrations were similar to those recorded in previous fatal isoflurane poisoning cases, and we concluded that the decedent had inhaled a high concentration of isoflurane using a plastic bag and died owing to acute toxicity resulting in respiratory failure. Tissue distribution patterns aligned with the lipophilic nature of isoflurane: adipose tissue had the highest isoflurane concentration (779.8 µg/mL), and the concentrations were higher in liver (194.2 µg/mL) and brain (149.0 µg/mL) tissues than in others. This case highlights a grave concern regarding the management of drugs that may be abused in veterinary clinics in Japan. Stricter controls and pharmacist involvement in veterinary medication management are needed to prevent the abuse of these drugs.

## Introduction

Isoflurane (1-chloro-2,2,2-trifluoroethyl difluoromethyl ether; boiling point 50 °C) is a halogenated, ether-based, volatile, inhalation anesthetic that is used for the induction and maintenance of general anesthesia in humans and animals [1, 2]. Isoflurane was first marketed in the United States in the 1980 s and was launched in Japan in the 1990 s [3, 4]. In recent years, the frequency of the use of isoflurane for humans has decreased owing to the spread of sevoflurane and desflurane, which produce anesthetic effects that are easier to control. However, isoflurane is still widely used in veterinary medicine [5].

Isoflurane is sometimes abused for recreational or suicidal purposes [6]. Because access to anesthetics such as isoflurane is usually limited to medical and research personnel, most abusers of these drugs also belong to these occupations [7–10]. While there are a few reports of isoflurane-related deaths, they did not show detailed case information and toxicological data, including blood and tissue concentrations [8, 10, 11]. Herein, we report a forensic autopsy case in which the deceased appeared to have died while abusing isoflurane supplied to a veterinary clinic. We measured the isoflurane concentrations in the blood and tissues using gas chromatography-mass spectrometry (GC–MS) to obtain information about the circumstances of the death.

## Case history

A man in his 40 s who worked at a veterinary hospital was found dead in the living room of his house by a family member. There was a bottle of isoflurane near him. Additionally, his left hand was holding a plastic bag, which contained a small amount of liquid, close to his mouth.

Six months before his death, he had been diagnosed with depression and insomnia and was prescribed sulpiride and lemborexant. His family had witnessed him sniffing isoflurane poured into a cup at home several times during the same period. He is thought to have no other history of drug abuse.

## Autopsy findings

**Fig. 1:**
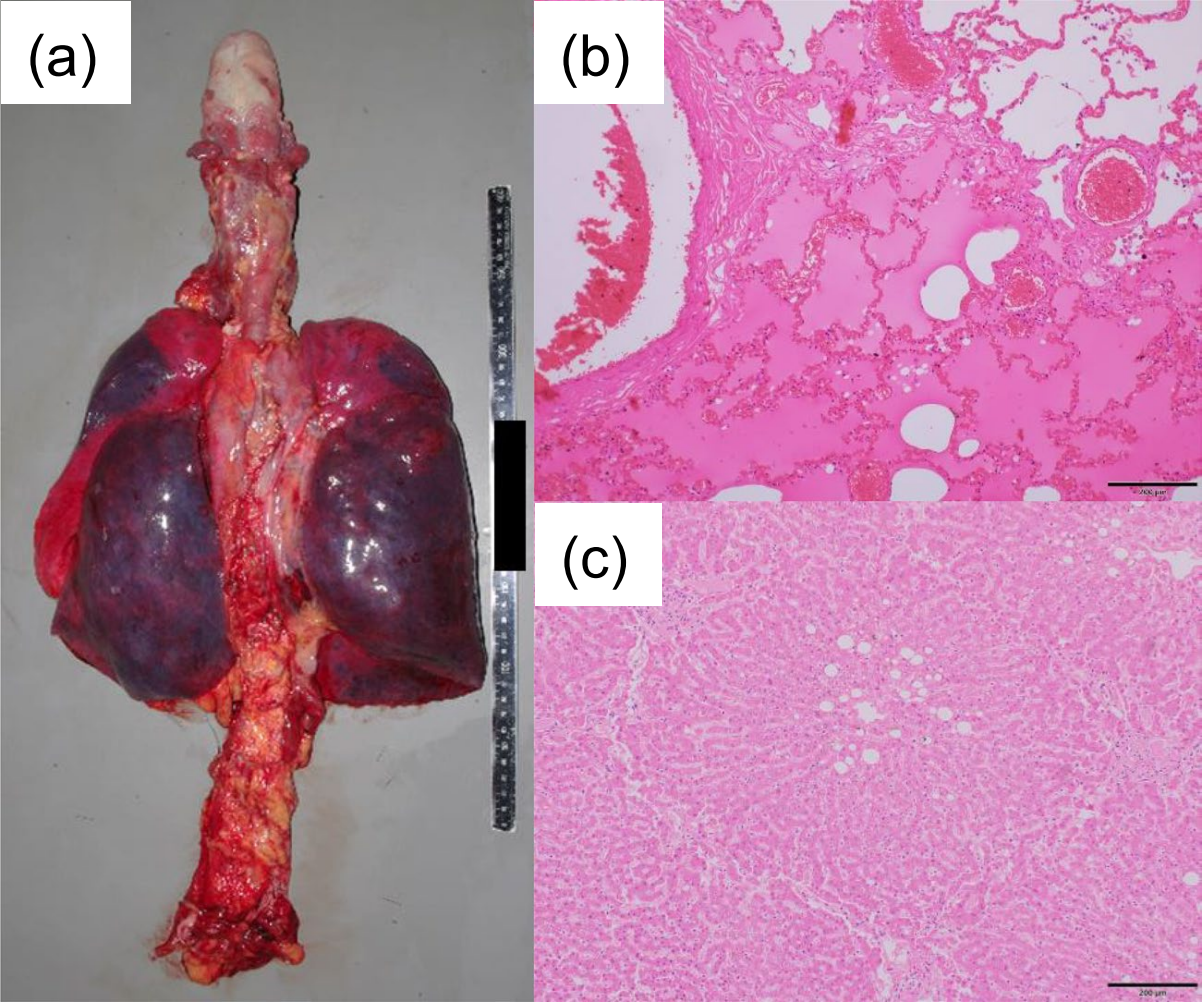
(a) Pulmonary edema (left lung: 556 g, right lung: 628 g). (b) Histological examination shows congestive pulmonary edema (HE). (c) Moderate fatty degeneration around the central hepatic vein (HE)

A forensic autopsy was performed 2 days after the body was found. The deceased was 173 cm tall and weighed 70.7 kg. There was partial subcutaneous hemorrhage without cranial fractures. The brain (1450 g) was congested, and the heart (370 g) contained dark red uncoagulated blood. There was pulmonary edema (left lung: 556 g, right lung: 628 g, Fig. 1a) and white mucus in the airways. Most organs were congested, including the liver (1508 g).

Pathological examination showed a congested cerebrum, congestive edema in the lungs (Fig. 1b), and moderate fatty degeneration around the central vein in the liver (Fig. 1c).

**Fig. 2:**
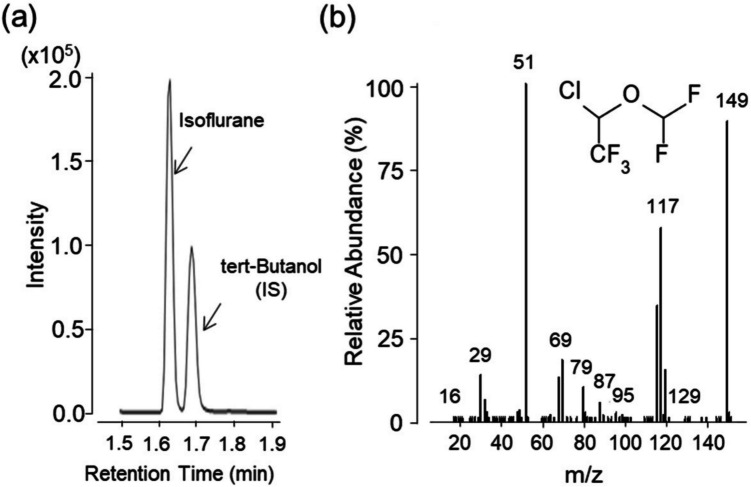
(a) Gas chromatography–mass spectrometry total ion current chromatogram of a routine scan for alcohol using the heart blood. There is no ethanol peak at the retention time of 1.86 min; however, the peak at the retention time of 1.62 min was identified as isoflurane. The peak detected at 1.70 min is tert-butanol as an internal standard of the analysis. (b) The mass chromatogram of the isoflurane peak at 1.62 min and the chemical structure of isoflurane

Routine GC–MS analysis for alcohol showed no ethanol in the blood. However, there was a peak at the retention time of 1.62 min that was identified as isoflurane (Fig. 2). Blood drug screening by liquid chromatography-tandem mass spectrometry detected therapeutic concentrations of sulpiride. Samples were collected and immediately stored at –30 °C until quantitative isoflurane analysis.

## Quantitative analysis of isoflurane

### Reagents

Isoflurane and dichloromethane (internal standard; IS) were purchased from FUJIFILM Wako Pure Chemical Corporation (Osaka, Japan). The dilution solvent tetraethylene glycol dimethyl ether (TEGDE) was purchased from Sigma-Aldrich (St Louis, MO, USA). All other chemicals were analytical grade and were purchased through local suppliers.

### Sample preparation

Liquid samples (left heart blood, right heart blood, femoral blood, urine, and gastric contents) and tissues samples (brain, lung, liver, kidney, adipose tissue, and muscle) were used for quantitative isoflurane analysis. Liquid samples were thawed, and frozen tissue samples were sliced to a thickness of approximately 0.1 cm before analysis. Sample vials (20 mL headspace glass vials) containing 0.95 mL of distilled water and 4 µL of 0.5 mg/mL dichloromethane-TEGDE solution (IS) had either 0.05 mL of blood or 0.05 g of tissue added and were then tightly sealed. For quantitative analysis, calibration standard vials of blank blood containing various concentrations of isoflurane-TEGDE solution (0, 10, 20, 50, 100, and 200 µg/mL) were also prepared as mentioned above. Sample vials and calibration standard vials were prepared in triplicate and tested. Only for the quantification of adipose tissue, blank blood containing higher concentrations of isoflurane-TEGDE solution (0, 400, 600, 800, 1000, and 1200 µg/mL) and 4 µL of 5.0 mg/mL dichloromethane-TEGDE solution (IS) were used for preparing calibration standard vials. Adipose tissue sample vials were prepared in quintuplicate and tested.

### GC–MS analysis

Headspace GC–MS analysis was performed using an Agilent G1888 network headspace sampler connected to a gas chromatograph-mass spectrometer (Agilent GC7890A, 5977MSD) (Agilent Technologies, Santa Clara, CA, USA). A DB-WAX capillary column was used (30 m × 0.25 mm id, 0.25 µm thickness; Agilent Technologies, Santa Clara, CA, USA). The headspace sampler conditions were as follows: vial incubation, 70 °C for 10 min; sample loop temperature, 120 °C; transfer line temperature, 120 °C. The gas chromatograph conditions were as follows: split injection mode (20:1); injector temperature, 150 °C; carrier gas, helium; flow rate, 1.5 mL/minute; oven temperature, 50–100 °C at a rate of 10 °C/minute. The mass spectrometer conditions were as follows: scan mode, *m/z* 45–300; ionization, electron ionization mode with an electron energy of 70 eV. In the mass spectrometry procedure, the scan mode was used for qualification and quantification.

### Method validation

A 5- or 6-point calibration curve was constructed by plotting the peak area ratio of isoflurane to the IS against isoflurane concentration, and fitting was performed via least-squares linear regression without a weighting factor. Precision (%) and accuracy (%) were determined by analyzing three control samples containing known amounts of isoflurane. Precision was expressed as the relative standard deviation of the concentration values. Accuracy was expressed as the percentage of differences between the observed concentrations and the expected concentrations. The limit of quantification was evaluated based on the precision data recorded below 20%.

### Quantitative analysis results

**Fig. 3:**
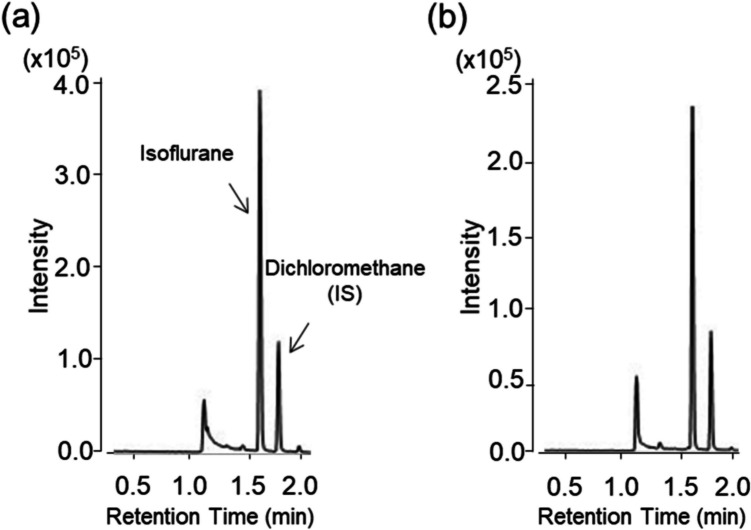
Gas chromatography–mass spectrometry total ion current chromatogram of the right heart blood (a) and a standard blood sample (50 µg/mL isoflurane) (b) in the quantitative analysis of isoflurane. The peak at 1.6 min is isoflurane, the peak at 1.8 min is dichloromethane as an internal standard for the analysis, and the peak at 1.2 min is air

Figure 3 shows the GC–MS total ion current chromatogram of the right heart blood and a standard blood sample (50 µg/mL isoflurane) in the quantitative analysis of isoflurane. The calibration curves for the quantification of isoflurane concentrations in the samples showed linearity in both concentrations between 20–200 µg/mL and 400–1200 µg/mL. Table 1 shows the validation data; the accuracy and precision were both acceptable at < 15%. Subsequently, the isoflurane concentrations in the samples of the present case were determined using this method.

**Table 1:** Validation data for the quantitative determination of isoflurane concentrations (*n* = 3)

Isoflurane concentration (µg/mL)		Accuracy (%)	Precision (%)
	20	–4.4	3.4
	50	4.2	10.6
	100	9.2	10.0
	200	–2.8	9.8
	400	3.3	3.1
	600	2.9	5.0
	800	2.0	1.5
	1000	–1.3	2.5
	1200	–0.9	1.1

Table 2 shows the isoflurane concentrations in the blood and tissue samples in this case and in previously reported isoflurane poisoning cases (Cases 1–3 [2, 8, 10]).

**Table 2:** Isoflurane concentrations (µg/mL or g) and other information for the present case and previously reported isoflurane poisoning cases

	Present case	Case 1 [8]	Case 2 [10]	Case 3 [10]
Left heart blood	49.8 ± 2.0	47.9 ± 0.94*	9.9*	45.9*
Right heart blood	54.6 ± 3.0			
Femoral blood	44.7 ± 4.0			
Urine	< 20	4.4 ± 0.14		
Gastric contents	44.2 ± 4.3	252.7 ± 16.05		
Brain	149.0 ± 14.9	306.9 ± 9.55	107.0	
Lung	33.7 ± 16.1		17.0	34.5
Liver	194.2 ± 29.4	999.8 ± 89.99	31.0	97.2
Kidney	34.6 ± 5.0	52.8 ± 4.70	22.3	27.3
Adipose tissue	779.8 ± 72.2			
Muscle	35.0 ± 21.8			
Other drugs detected in blood	Sulpiride	Nordazepam	Diphenhydramine, Orphenadrine	
Type of isoflurane usage	Recreational	Recreational	Recreational	Suicidal
Use of a plastic bag for inhalation	Yes	Yes	No	Yes

^*^Cardiac blood was analyzed in these cases

## Discussion

In accordance with the pharmacokinetics of isoflurane, the isoflurane concentration in arterial blood rapidly increases when the gas is inhaled by healthy adults [12–14]. A prior study showed that in healthy adults, inhalation of 1.2% (n = 7) or 1.8% (n = 6) isoflurane for 1 h resulted in a rapid increase in arterial blood isoflurane concentrations to mean values of 71 µg/mL and 101 µg/mL, respectively [12]. Ten minutes after the discontinuation of isoflurane inhalation, the mean arterial concentrations had rapidly decreased to 17 µg/mL and 29 µg/mL, respectively [12]. The elimination half-life was reported to be biphasic, with a first-phase half-life of 2.2–2.8 min and a second-phase half-life of 50.2–51.0 min [12]. In the current case, the isoflurane concentrations in the left and right heart blood and femoral blood were 49.8, 54.6, and 44.7 µg/mL, respectively. These concentrations were slightly lower than the isoflurane blood concentrations seen in surgical procedures with ventilator-assisted respiratory management (71–101 µg/mL) [12]. As isoflurane strongly depresses respiration and muscular activity [15], the isoflurane concentrations in the present case likely caused fatal asphyxiation in the absence of artificial ventilation. The cardiac blood concentrations in the previously reported Case 1 (47.9 µg/mL [8]) and Case 3 (45.9 µg/mL [10]) are similar to those reported in the present case. The decedents in the current case, Case 1 [8], and Case 3 [10] had a plastic bag near the face for isoflurane inhalation, while the decedent in Case 2 (cardiac blood concentration, 9.9 µg/mL [10]) had a bottle in his hand. Considering the autopsy findings, the isoflurane concentrations in the samples, and the pharmacokinetic properties of isoflurane, we concluded that the decedent in the current case would have inhaled a high concentration of isoflurane using a plastic bag and died owing to acute toxicity resulting in respiratory failure. Additionally, the moderately fatty liver in the present case may indicate habitual isoflurane use [16].

The urinary isoflurane concentration in the current case was lower than 20 µg/mL, which was the lower limit of determination. The urinary concentration in Case 1 [8], which has a similar cardiac blood concentration, was 4.4 µg/mL. Previous research has shown that 92.3% of isoflurane in the body is excreted from the lungs in exhaled air, while only 0.43% of isoflurane is excreted in the urine [17], as shown by the low urine concentration of isoflurane in the current case. The isoflurane concentration in the gastric contents (44.2 µg/mL) was almost the same as that in the blood, and may indicate that the decedent had not orally ingested isoflurane. The tissue distribution pattern of isoflurane in the present case was consistent with the characteristics of lipophilic compounds; the isoflurane concentration was highest in the adipose tissue, followed by the liver and brain. In Case 1, the highest isoflurane concentration was in the liver, followed by the brain; this pattern was generally consistent with our findings. Data for the isoflurane concentration in adipose tissue were not available for Cases 1–3. In Case 2, the isoflurane concentration was highest in the brain, followed by the liver. In Case 2, the cause of death was attributed to other drugs, and the duration of isoflurane inhalation may have been shorter than in the present case. Consequently, the isoflurane tissue distribution in Case 2 may have differed from that observed in the present case and in Case 1. Because of the limited number of studies reporting detailed tissue isoflurane concentrations and other such histological findings, especially the degree of fatty degradation of the liver, further comparisons were not possible.

Several limitations should be considered when interpreting the data in the present case. First, this report describes a single case, which restricts the extent to which general conclusions can be drawn. Second, only three reported cases of fatal isoflurane poisoning were available for comparison. Third, the postmortem interval likely influences the drug concentrations, particularly for lipophilic and volatile compounds such as isoflurane, which are highly susceptible to postmortem redistribution. Because the postmortem interval was not specified in the previously reported cases (Cases 1–3), direct comparisons of blood and tissue isoflurane concentrations between those cases and the present case should be interpreted with caution.

From the standpoint of social medicine, the present case highlights problems with the management of isoflurane in veterinary clinics. The deceased was still involved in the handling of isoflurane on the job, even after his abuse had been revealed. While there are strict laws regarding the handling of veterinary medicinal products in Japan, veterinary prescriptions are managed directly by veterinarians, unlike in many Western nations, with limited involvement of pharmacists [18, 19]. Recently, it has been realized that veterinary clinics may be a source of opioids and other drugs of abuse [20]. To prevent the misuse of veterinary drugs, as exemplified by the present case, greater participation of pharmacists in the management and administration of veterinary pharmaceuticals should be actively promoted.

## Key points

1. This case highlights issues regarding the management of veterinary drugs in Japan.
2. A veterinary hospital staff member was found dead due to suspected isoflurane poisoning.
3. Blood and tissue isoflurane concentrations were quantitated by gas chromatography-mass spectrometry.
4. The cause of death was confirmed as acute isoflurane poisoning by inhalation.
5. The deceased had free access to all the veterinary drugs.

## Abbreviations

GC-MS: Gas chromatography-mass spectrometry
IS: Internal standard
TEGDE: Tetraethylene glycol dimethyl ether
